# 2-(Dihydroxy­meth­yl)pyridinium chloride

**DOI:** 10.1107/S1600536810006604

**Published:** 2010-02-27

**Authors:** Hui-Fen Qian, Wei Huang

**Affiliations:** a College of Sciences, Nanjing University of Technology, Nanjing 210009, People’s Republic of China; b State Key Laboratory of Coordination Chemistry, Nanjing National Laboratory of Microstructures, School of Chemistry and Chemical Engineering, Nanjing University, Nanjing 210093, People’s Republic of China

## Abstract

In the title compound, C_6_H_8_NO_2_^+^·Cl^−^, inter­molecular O—H⋯Cl and N—H⋯Cl hydrogen bonds are observed in which each chloride anion links three adjacent cations into a hydrogen-bond network.

## Related literature

For a related compound, see Mantero *et al.* (2006 ▶).
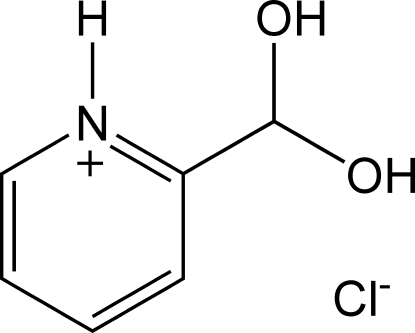


## Experimental

### 

#### Crystal data

C_6_H_8_NO_2_^+^·Cl^−^
*M* *_r_* = 161.58
Monoclinic, 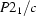

*a* = 4.6879 (7) Å
*b* = 15.557 (2) Å
*c* = 10.1199 (14) Å
β = 91.181 (2)°
*V* = 737.88 (18) Å^3^
*Z* = 4
Mo *K*α radiation
μ = 0.45 mm^−1^
*T* = 291 K
0.12 × 0.12 × 0.10 mm

#### Data collection

Bruker SMART 1K CCD area-detector diffractometer
Absorption correction: multi-scan (*SADABS*; Bruker, 2000 ▶) *T*_min_ = 0.948, *T*_max_ = 0.956
3676 measured reflections
1303 independent reflections
842 reflections with *I* > 2σ(*I*)
*R*_int_ = 0.058

#### Refinement

*R*[*F* ^2^ > 2σ(*F* ^2^)] = 0.035
*wR*(*F* ^2^) = 0.075
*S* = 0.89
1303 reflections
99 parameters
2 restraints
H atoms treated by a mixture of independent and constrained refinement
Δρ_max_ = 0.22 e Å^−3^
Δρ_min_ = −0.23 e Å^−3^

### 

Data collection: *SMART* (Bruker, 2000 ▶); cell refinement: *SAINT* (Bruker, 2000 ▶); data reduction: *SAINT*; program(s) used to solve structure: *SHELXTL* (Sheldrick, 2008 ▶); program(s) used to refine structure: *SHELXTL*; molecular graphics: *SHELXTL* software used to prepare material for publication: *SHELXTL*.

## Supplementary Material

Crystal structure: contains datablocks global, I. DOI: 10.1107/S1600536810006604/bg2326sup1.cif

Structure factors: contains datablocks I. DOI: 10.1107/S1600536810006604/bg2326Isup2.hkl

Additional supplementary materials:  crystallographic information; 3D view; checkCIF report

## Figures and Tables

**Table 1:** Hydrogen-bond geometry (Å, °)

*D*—H⋯*A*	*D*—H	H⋯*A*	*D*⋯*A*	*D*—H⋯*A*
O2—H2*A*⋯Cl1^i^	0.85 (1)	2.24 (1)	3.089 (2)	176 (2)
O1—H1*A*⋯Cl1^ii^	0.85 (1)	2.19 (1)	3.0374 (18)	177 (3)
N1—H1⋯Cl1^iii^	0.86	2.33	3.115 (2)	151

Symmetry codes: (i) 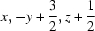; (ii) 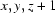; (iii) 
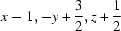
.

**Fig. 1.**
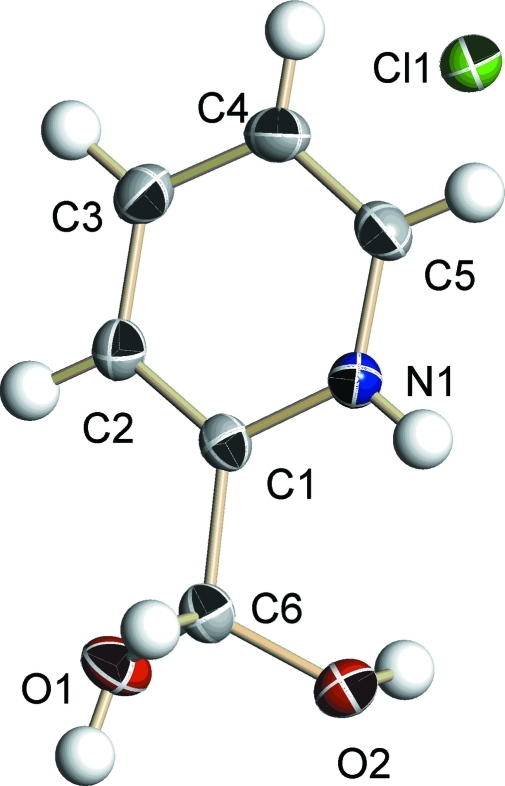
The molecular structure of the title compound with the atom-numbering scheme. Displacement ellipsoids are drawn at the 30% probability level.

**Fig. 2.**
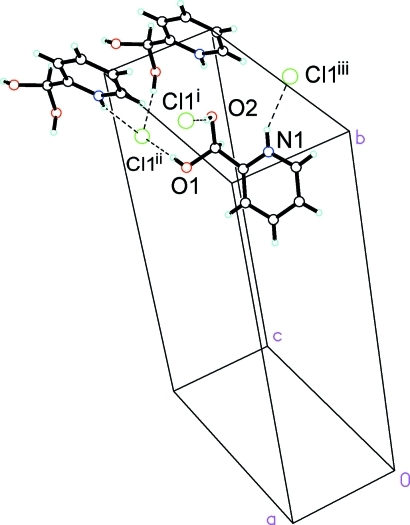
Perspective view of the hydrogen bonding interactions in the crystal packing of (I), where the hydrogen bonds are shown as dashed lines. [Symmetry codes: (i) x - 1, -y+3/2, z + 1/2; (ii) x, y1/2, z + 1; (iii) x, -y+3/2, z + 1/2.]

## supplementary crystallographic information

### Comment

The crystal structure of pyridin-4-ylmethanediol, namely the hydrated form of isonicotinaldehyde has been previously reported (Mantero *et al.*, 2006). In this paper, we report the X-ray single-crystal structure of pyridin-2-ylmethanediol-1-ium chloride (I).

The molecular structure of (I) is illustrated in Fig. 1. The two hydroxyl groups lie at the same side of the aromatic ring. In the crystal packing, intermolecular O—H···Cl and N—H···Cl hydrogen bonding interactions are observed where every chloride anion links three adjacent molecules into a hydrogen-bond sustained network (Fig. 2).

### Refinement

The H1A atom bonded with atom O1 was located in the difference synthesis and were refined isotropically. The other H atoms were placed in geometrically idealized positions and refined as riding, with C—H = 0.93–0.98 Å, N—H = 0.86 Å and O—H = 0.96 Å, *U*_iso_(H) = 1.2*U*_eq_(C), *U*_iso_(H) = 1.2*U*_eq_(N) and *U*_iso_(H) = 1.5*U*_eq_(O).

### Crystal data

**Table:**

C_6_H_8_NO_2_^+^·Cl^−^	*F*(000) = 336
*M**_r_* = 161.58	*D*_x_ = 1.455 Mg m^−^^3^
Monoclinic, *P*2_1_/*c*	Mo *K*α radiation, λ = 0.71073 Å
Hall symbol: -P 2ybc	Cell parameters from 776 reflections
*a* = 4.6879 (7) Å	θ = 2.4–21.0°
*b* = 15.557 (2) Å	µ = 0.45 mm^−^^1^
*c* = 10.1199 (14) Å	*T* = 291 K
β = 91.181 (2)°	Block, colourless
*V* = 737.88 (18) Å^3^	0.12 × 0.12 × 0.10 mm
*Z* = 4	

### Data collection

**Table:**

Bruker SMART 1K CCD area-detector diffractometer	1303 independent reflections
Radiation source: fine-focus sealed tube	842 reflections with *I* > 2σ(*I*)
graphite	*R*_int_ = 0.058
φ and ω scans	θ_max_ = 25.0°, θ_min_ = 2.4°
Absorption correction: multi-scan (*SADABS*; Bruker, 2000)	*h* = −5→5
*T*_min_ = 0.948, *T*_max_ = 0.956	*k* = −12→18
3676 measured reflections	*l* = −12→10

### Refinement

**Table:**

Refinement on *F*^2^	Primary atom site location: structure-invariant direct methods
Least-squares matrix: full	Secondary atom site location: difference Fourier map
*R*[*F*^2^ > 2σ(*F*^2^)] = 0.035	Hydrogen site location: inferred from neighbouring sites
*wR*(*F*^2^) = 0.075	H atoms treated by a mixture of independent and constrained refinement
*S* = 0.89	*w* = 1/[σ^2^(*F*_o_^2^) + (0.0272*P*)^2^] where *P* = (*F*_o_^2^ + 2*F*_c_^2^)/3
1303 reflections	(Δ/σ)_max_ = 0.001
99 parameters	Δρ_max_ = 0.22 e Å^−^^3^
2 restraints	Δρ_min_ = −0.23 e Å^−^^3^

### Special details

**Table:**

Experimental. The structure was solved by direct methods (Bruker, 2000) and successive difference Fourier syntheses.
Geometry. All esds (except the esd in the dihedral angle between two l.s. planes) are estimated using the full covariance matrix. The cell esds are taken into account individually in the estimation of esds in distances, angles and torsion angles; correlations between esds in cell parameters are only used when they are defined by crystal symmetry. An approximate (isotropic) treatment of cell esds is used for estimating esds involving l.s. planes.
Refinement. Refinement of *F*^2^ against ALL reflections. The weighted *R*-factor *wR* and goodness of fit *S* are based on *F*^2^, conventional *R*-factors *R* are based on *F*, with *F* set to zero for negative *F*^2^. The threshold expression of *F*^2^ > σ(*F*^2^) is used only for calculating *R*-factors(gt) *etc*. and is not relevant to the choice of reflections for refinement. *R*-factors based on *F*^2^ are statistically about twice as large as those based on *F*, and *R*- factors based on ALL data will be even larger.

### Fractional atomic coordinates and isotropic or equivalent isotropic displacement parameters (Å^2^)

**Table:**

	*x*	*y*	*z*	*U*_iso_*/*U*_eq_	
C1	0.0342 (4)	0.62701 (15)	0.8930 (2)	0.0395 (6)	
C2	0.1167 (5)	0.54999 (16)	0.8429 (2)	0.0469 (6)	
H2	0.2515	0.5168	0.8883	0.056*	
C3	0.0002 (5)	0.52104 (16)	0.7246 (2)	0.0544 (7)	
H3	0.0549	0.4681	0.6906	0.065*	
C4	−0.1975 (5)	0.57099 (17)	0.6572 (2)	0.0553 (7)	
H4	−0.2792	0.5519	0.5780	0.066*	
C5	−0.2716 (5)	0.64860 (17)	0.7082 (2)	0.0504 (7)	
H5	−0.4015	0.6836	0.6630	0.060*	
C6	0.1322 (5)	0.66291 (15)	1.0257 (2)	0.0448 (6)	
H6	0.0102	0.6382	1.0935	0.054*	
Cl1	0.51126 (13)	0.65293 (4)	0.34503 (6)	0.0572 (2)	
H1A	0.442 (6)	0.6405 (18)	1.1316 (11)	0.090 (11)*	
H2A	0.211 (5)	0.7784 (16)	0.978 (2)	0.080 (11)*	
N1	−0.1571 (4)	0.67433 (12)	0.82335 (17)	0.0424 (5)	
H1	−0.2077	0.7233	0.8546	0.051*	
O1	0.4092 (3)	0.63308 (12)	1.04924 (17)	0.0573 (5)	
O2	0.1066 (4)	0.75152 (12)	1.03217 (17)	0.0567 (5)	

### Atomic displacement parameters (Å^2^)

**Table:**

	*U*^11^	*U*^22^	*U*^33^	*U*^12^	*U*^13^	*U*^23^
C1	0.0371 (13)	0.0428 (15)	0.0389 (13)	−0.0011 (11)	0.0022 (11)	0.0061 (11)
C2	0.0486 (14)	0.0414 (15)	0.0508 (15)	0.0054 (12)	0.0005 (12)	0.0027 (12)
C3	0.0671 (17)	0.0424 (16)	0.0537 (17)	−0.0014 (14)	0.0030 (14)	−0.0052 (13)
C4	0.0661 (18)	0.0557 (18)	0.0439 (15)	−0.0126 (15)	−0.0050 (13)	−0.0025 (13)
C5	0.0529 (16)	0.0540 (17)	0.0441 (15)	−0.0023 (13)	−0.0062 (12)	0.0083 (13)
C6	0.0431 (14)	0.0467 (16)	0.0446 (14)	0.0032 (13)	0.0011 (11)	0.0031 (12)
Cl1	0.0683 (5)	0.0469 (4)	0.0559 (4)	−0.0043 (3)	−0.0107 (3)	−0.0036 (3)
N1	0.0464 (12)	0.0389 (12)	0.0418 (12)	0.0010 (10)	0.0008 (9)	0.0013 (9)
O1	0.0480 (11)	0.0753 (14)	0.0483 (12)	0.0133 (9)	−0.0086 (9)	−0.0063 (9)
O2	0.0670 (13)	0.0478 (12)	0.0551 (12)	0.0021 (10)	−0.0013 (10)	−0.0084 (9)

### Geometric parameters (Å, °)

**Table:**

C1—N1	1.348 (3)	C5—N1	1.334 (3)
C1—C2	1.360 (3)	C5—H5	0.9300
C1—C6	1.518 (3)	C6—O2	1.385 (3)
C2—C3	1.381 (3)	C6—O1	1.395 (3)
C2—H2	0.9300	C6—H6	0.9800
C3—C4	1.379 (3)	N1—H1	0.8600
C3—H3	0.9300	O1—H1A	0.852 (10)
C4—C5	1.361 (3)	O2—H2A	0.853 (10)
C4—H4	0.9300		
			
N1—C1—C2	118.5 (2)	N1—C5—H5	120.1
N1—C1—C6	116.6 (2)	C4—C5—H5	120.1
C2—C1—C6	124.8 (2)	O2—C6—O1	113.84 (19)
C1—C2—C3	120.0 (2)	O2—C6—C1	112.52 (18)
C1—C2—H2	120.0	O1—C6—C1	106.99 (18)
C3—C2—H2	120.0	O2—C6—H6	107.7
C4—C3—C2	119.6 (2)	O1—C6—H6	107.7
C4—C3—H3	120.2	C1—C6—H6	107.7
C2—C3—H3	120.2	C5—N1—C1	123.0 (2)
C5—C4—C3	119.1 (2)	C5—N1—H1	118.5
C5—C4—H4	120.4	C1—N1—H1	118.5
C3—C4—H4	120.4	C6—O1—H1A	105.8 (19)
N1—C5—C4	119.7 (2)	C6—O2—H2A	114.0 (19)
			
N1—C1—C2—C3	−1.4 (3)	C2—C1—C6—O2	157.6 (2)
C6—C1—C2—C3	175.9 (2)	N1—C1—C6—O1	−150.91 (18)
C1—C2—C3—C4	0.6 (4)	C2—C1—C6—O1	31.8 (3)
C2—C3—C4—C5	0.8 (4)	C4—C5—N1—C1	0.7 (3)
C3—C4—C5—N1	−1.4 (4)	C2—C1—N1—C5	0.7 (3)
N1—C1—C6—O2	−25.2 (3)	C6—C1—N1—C5	−176.7 (2)

### Hydrogen-bond geometry (Å, °)

**Table:**

*D*—H···*A*	*D*—H	H···*A*	*D*···*A*	*D*—H···*A*
O2—H2A···Cl1^i^	0.85 (1)	2.24 (1)	3.089 (2)	176 (2)
O1—H1A···Cl1^ii^	0.85 (1)	2.19 (1)	3.0374 (18)	177 (3)
N1—H1···Cl1^iii^	0.86	2.33	3.115 (2)	151

Symmetry codes: (i) *x*, −*y*+3/2, *z*+1/2; (ii) *x*, *y*, *z*+1; (iii) *x*−1, −*y*+3/2, *z*+1/2.
